# Downregulated Expression of hsa_circ_0005556 in Gastric Cancer and Its Clinical Significance

**DOI:** 10.1155/2019/2624586

**Published:** 2019-11-19

**Authors:** Liangwei Yang, Yu Yu, Xiuchong Yu, Jiaming Zhou, Zhiping Zhang, Shibo Ying, Junming Guo, Zhilong Yan

**Affiliations:** ^1^Medical School of Ningbo University, Ningbo No. 1 Hospital, Department of Biochemistry and Molecular Biology, Zhejiang Key Laboratory of Pathophysiology, Ningbo 315211, China; ^2^Department of Gastrointestinal Surgery, Ningbo No. 1 Hospital, Ningbo 315010, China; ^3^Institute of Occupational Diseases, Zhejiang Academy of Medical Sciences, Hangzhou 310013, China

## Abstract

**Background:**

Gastric cancer (GC) has a poor prognosis due to the lack of ideal tumor markers. Circular RNAs (circRNAs) are a novel type of noncoding RNA related to the occurrence of GC. Among our research, we investigated the role of hsa_circ_0005556 in GC.

**Materials and Methods:**

The expression of hsa_circ_0005556 of 100 paired GC tissues and adjacent normal tissues was detected using quantitative reverse transcription-polymerase chain reaction (qRT-PCR). A receiver operating characteristic (ROC) curve was established to evaluate the diagnostic value of hsa_circ_0005556. The correlation between the expression of hsa_circ_0005556 and corresponding clinicopathological characteristic was explored.

**Results:**

hsa_circ_0005556 was significantly downregulated in GC tissues contrasted with adjacent normal tissues (*n* = 100, *p* < 0.001). The areas under the ROC curve (AUC) of hsa_circ_0005556 were up to 0.773, while 64% sensitivity and 82% specificity, respectively. Moreover, its expression levels were significantly associated with differentiation (*p* = 0.001), TNM stage (*p* = 0.013), and lymphatic metastasis (*p* = 0.039). GC patients of high hsa_circ_0005556 levels had a longer overall survival (OS) than those of the low group (*p* = 0.047).

**Conclusion:**

hsa_circ_0005556 is a potential biomarker for GC, which may guide judgment of the indication of endoscopic treatment for early gastric cancer (EGC).

## 1. Introduction

Gastric cancer (GC), a serious global health risks, ranks as the fifth most common cancer and the third primary cause of cancer-related death globally. Every year, beyond 1000000 new cases and 783000 new deaths are reported [1]. Incidence rates are markedly elevated in Eastern Asia [1]. The prognosis of GC is closely associated with the stage when diagnosed. The 5-year survival rate of stage I patients can reach 90%, while that of the stage IV patients who already show distant metastases is only approximately 14% [2]. Most patients with early gastric cancer (EGC) have no obvious specific symptoms [3]. The diagnosis of GC relies on upper gastrointestinal (GI) endoscopy. However, in most countries, upper GI endoscopy is not a routine medical examination [4]. Most GC patients are diagnosed at an advanced stage. On a global scale, the 5-year survival rate of GC is an average of 20-40% [5]. Therefore, to improve the diagnosis of EGC, it is critical to find potent, stable, and ideal diagnostic biomarkers of GC.

With the development of RNA high-throughput sequencing technology and advances in biophysical technology, an increasing number of noncoding RNAs have been discovered [6]. Some studies have found that noncoding RNA is involved in the pathogenesis of many cancers [7, 8]. Circular RNAs (circRNAs) are a novel type of endogenous noncoding RNAs produced by precursor mRNA backsplicing using exons, introns, or intergenic regions [9]. In contrast with linear RNAs, circRNAs are discriminated by a circle structure linked by a covalent bond and neither a 5′ cap structure nor a 3′ polyadenylated tail. As a result of the circular structure, circRNAs are more stable, conserved, and highly abundant than other noncoding RNAs, such as miRNAs and lncRNAs [10–13]. Circular RNA_LARP4 was proved to downregulate in GC tissues and correlated to good survival [14]. It inhibited GC cell proliferation and invasion via sponging hsa-miR-424-5p and regulating the expression of tumor suppressor kinase 1 (LATS1). Another study indicated that the level of circPVT1 is elevated in GC samples and predicted favorable survival of patients with GC. circPVT1 acts as a sponge for members of the miR-125 family to promote the proliferation of GC cell [15]. Due to these biological properties, circRNAs may be important biomarkers for the occurrence and development of GC.

Based on our previous microarray analysis (GEO No. GSE89143, Guo, 2016: https://www.ncbi.nlm.nih.gov/geo/query/acc.cgi?acc=GSE89143), the expression of hsa_circ_0005556 was quite different in GC tissue compare to paired normal tissue. So, we chose hsa_circ_0005556 as a research target to analyze its diagnostic values of GC. The gene of hsa_circ_0005556 is located at chr2:15693549-15698758. Its spliced sequence length is 218 nt, and the related gene symbol is the neuroblastoma-amplified sequence (NBAS). To our knowledge, few studies have identified the functions of hsa_circ_0005556. Here, quantitative reverse transcription-polymerase chain reaction (qRT-PCR) was utilized to explore the expression of hsa_circ_0005556 in GC patients. And the relationship with clinicopathological factors was also analyzed. We identified hsa_circ_0005556 as a diagnostic biomarker in GC.

## 2. Materials and Methods

### 2.1. Samples and Clinical Information

GC tissue samples were obtained from Ningbo Yinzhou People's Hospital from January 2010 to December 2015. During this time, all specimens were obtained from GC patients undergoing gastroenterology and collected by the surgeons. GC tissues were taken from the mucosa of the center of the tumor. The adjacent normal tissues were taken from the mucosa 5 cm beyond the edge of carcinoma. All fresh samples were instantly stored in RNA-fixer Reagent (BioTeke, Beijing, China), then preserved at -80°C until RNA isolation.

The clinical information of all samples was collected. The GC diagnosis was confirmed by histopathology. The pathologic stage of the tumor was assigned basing on the tumor-node metastasis (TNM) staging system of the International Union Against Cancer (8^th^ edition). The histological grade of the tumor was assigned basing on the American Joint Committee on Cancer (AJCC) cancer staging manual (8^th^ edition). Written informed consent was acquired from all individuals. This research acquired the approval from the Human Research Ethics Committee of Ningbo University (IRB No. 20100303).

### 2.2. Total RNA Extraction and Quality Control

According to the manufacturer's instructions, total RNA of tissues was extracted with TRIzol reagent (Invitrogen, Karlsruhe, Germany). Next, the DS-11 spectrophotometer machine (DeNovix, Wilmington, DE, USA) was used to verify purity and concentration of total RNA. The A260/A280 ratio was used to evaluate the RNA purity, which only ranged from 1.8 to 2.0 for the samples that were qualified and used.

### 2.3. Reverse Transcription

Total RNA was performed to synthesize cDNA using the GoScript RT System (Promega, Madison, WI, USA) with a random primer, following the manufacturer's instructions.

### 2.4. Quantitative Reverse Transcription-Polymerase Chain Reaction

qRT-PCR analysis was implemented on an Mx3005P real-time PCR System (Stratagene, La Jolla, CA, USA) with GoTaq qPCR Master Mix (Promega). The sequences of divergent primers were designed and produced by BGI (Shenzhen, China) Co., Ltd. The primer of hsa_circ_0005556 was 5′-GTGTGTGGAAATCAGCCTAG-3′ and 5′-AACCAAGCGAACCAGTCCAT-3′. The primer of glyceraldehyde 3-phosphate dehydrogenase (GAPDH, as control) primers was 5′-CTGCCAACGTGTCAGTGGTG-3′ and 5′-TCAGTGTAGCCCAGGATGCC-3′. The conditions of thermal cycling were as follows: hot start at 95°C for 5 minutes, next 40 cycles at 95°C for 15 seconds, 56°C for 30 seconds, and 72°C for 30 seconds. The qRT-PCR products were confirmed by Sanger sequencing, which was completed by Geneseed (Guangzhou, China). The relative quantification level of circRNA was calculated by the *Δ*Cq (quantification cycle) method. A larger *Δ*Cq indicates a lower level. Through three independent experiments, all of the data were expressed as the mean ± SD.

### 2.5. Statistical Analysis

Statistical data were analyzed by Statistical Product and Service Solutions (SPSS) 19.0 software (SPSS, Chicago, IL, USA). Figures and tables were produced by GraphPad Prism 5.0 (GraphPad Software, La Jolla, CA, USA). The data in this paper are in accordance with the normal distribution. The Shapiro-Wilk test, Student's *t*-test, analysis of variance (ANOVA), receiver operating characteristic (ROC) curves, and Kaplan-Meier survival curves were properly used. A value of *p* < 0.05 (two sided) was considered to be statistically significant.

## 3. Results

### 3.1. The Expression of hsa_circ_0005556 in GC Tissues

**Figure 1:**
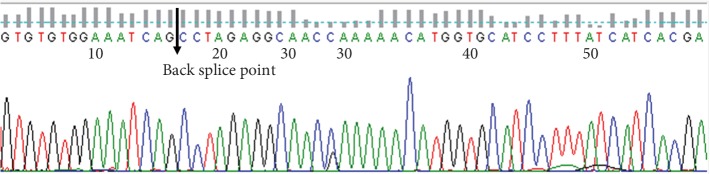
Sanger sequence results of qRT-PCR products of hsa_circ_0005556 in gastric cancer tissues.

**Figure 2:**
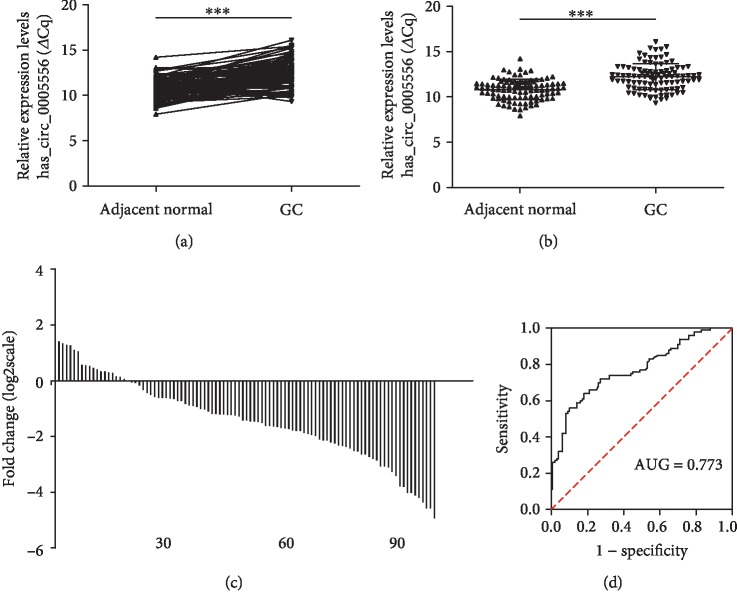
The expression of hsa_circ_0005556 in gastric cancer tissue. Statistical significance was defined as two-sided ^∗∗∗^<0.001. (a) The expression level of hsa_circ_0005556 in gastric cancer tissues (*n* = 100) and corresponding adjacent normal tissues (*n* = 100). (b) The expression levels of hsa_circ_0005556 were significantly lower than those in adjacent normal tissues (*n* = 100, *p* < 0.001). (c) The percentage of low expression of hsa_circ_0005556 in tissue accounts for 81% (81/100). (d) The ROC curve of hsa_circ_0005556. The area under the curve was up to 0.773.

To detect the expression of hsa_circ_0005556 in GC tissues, qRT-PCR was used in 100 pairs of GC and adjacent normal samples. As shown by Sanger sequencing, the sequence of the PCR products of hsa_circ_0005556 contains the cyclization site (Figure 1). The difference in *Δ*Cq between cancer and adjacent normal tissues was in accordance with the normal distribution. The results indicated that hsa_circ_0005556 was significantly downregulated in GC tissues contrast to adjacent normal tissues (*n* = 100, *p* < 0.001, Figures 2(a) and 2(b)). Among all samples, samples with decreased expression accounted for 81.4% (Figure 2(c)).

### 3.2. Prediction of hsa_circ_0005556 Binding to miRNAs by Sequence Analysis

Many circRNAs have complementary sites with microRNA (miRNAs) called microRNA response elements. Given that miRNAs function as downstream regulatory elements of circRNAs to affect the progression of GC, identification of the signal axis from circRNAs to miRNAs is important. We predicted the potential binding miRNAs for hsa_circ_0005556 using sequence analysis.

The results showed there were 6 potential binding miRNAs (Table 1). According to these predicted circRNA-miRNA sponging sites, hsa_circ_0005556 may bind certain miRNAs, and their verified functions indicated a potential role involved in the adjustment of GC progression.

### 3.3. Diagnostic and Prognostic Value of hsa_circ_0005556 in GC

Since we discovered hsa_circ_0005556 was significantly downregulated in GC tissues, we further analyzed the connection between the expression of hsa_circ_0005556 and some clinicopathological characteristics of patients with GC. As described in Table 2, the hsa_circ_0005556 level in tissues were significantly correlated with differentiation (*p* = 0.001), TNM stage (*p* = 0.013), and lymphatic metastasis (*p* = 0.039). Besides, no relationship was detected between its level and the rest of clinicopathological factors, like age, gender, tumor diameter, invasion, distal metastasis, carbohydrate antigen 19-9 (CA19-9), and carcinoembryonic antigen (CEA).

**Figure 3:**
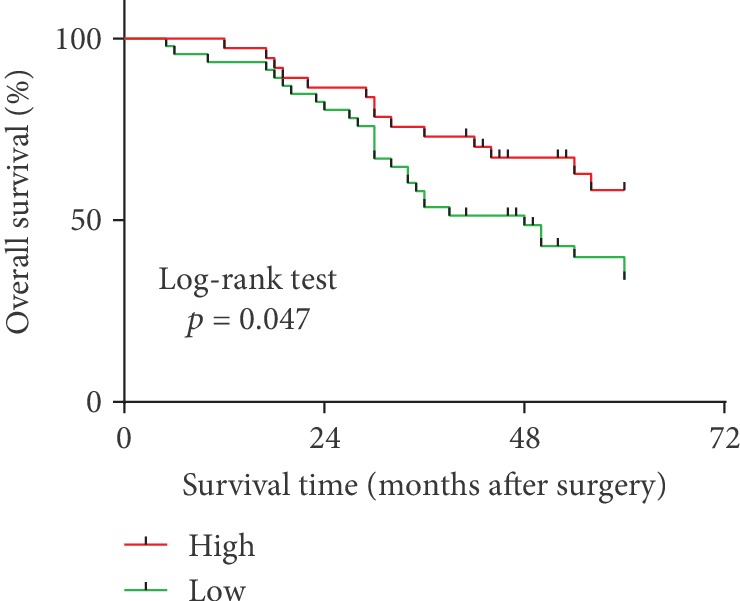
Kaplan-Meier survival analyses of overall survival (OS) based on the hsa_circ_0005556 level in GC tissues. OS time of the low hsa_circ_0005556 expression group was shorter than those of the high expression group (*p* = 0.047).

Next, the Kaplan-Meier survival analysis was conducted to analyze the overall survival (OS) of GC patients based on hsa_circ_0005556 expression. GC patients were divided into two groups, the low or high group according to the mean value of hsa_circ_0005556 expression in GC tissues. The results showed that patients in the low group had shorter OS compared to those in the high group (*p* = 0.047, Figure 3).

A ROC curve was established to test the potential diagnostic value of hsa_circ_0005556 (Figure 2(d)). The area under the ROC curve (AUC) reached 0.773, while 64% sensitivity and 82% specificity, respectively. The likelihood value was up to 3.56. Taken together, our research forecasted that hsa_circ_0005556 might act as an ideal biomarker for detecting GC.

## 4. Discussion

circRNAs were mistakenly considered to be aberrant splicing byproducts when they were discovered in viruses 40 years ago [29, 30]. Due to the revolutionary breakthrough in RNA-sequencing technique and biophysics techniques, many circRNAs have been discovered and were shown to be stable, conserved, and highly abundant [31, 32]. Due to these characteristics, circRNAs have certain advantages over other noncoding RNAs in the diagnosis and treatment of diseases.

The prognosis of GC is tightly associated with disease stage. Later stages implied a poor 5-year survival rate [33]. Thus, establishment of an early cancer screening system is important. circRNAs are acting in the regulation of cancer progression. In our research, contrast to adjacent normal tissues, hsa_circ_0005556 was downregulated in GC samples (Figure 2). Moreover, GC patients of a high hsa_circ_0005556 level in GC tissues showed better outcomes than those of a low level. These results indicated that hsa_circ_0005556 act a valuable role in both diagnosis and prognosis, which may be a potential biomarker that contributes to GC screening.

In recent years, a number of studies have focused on finding ideal biomarkers. Compared with another study on hsa_circ_0006633, whose specificity and sensitivity are 81% and 60%, respectively [34], hsa_circ_0005556 has a more precise diagnostic value (Figure 2(d)). hsa_circ_0006633 is associated with distant metastasis of GC (Table 1), suggesting long-term monitoring of GC recurrence. Different circRNAs play different roles in the management of GC.

The functions of circRNAs have not been fully revealed. Jeck et al. proposed the theory of miRNA sponges [11], in which circRNAs have miRNA response elements to serve on competing endogenous RNAs. We predicted 6 potential miRNA-binding sites of hsa_circ_0005556 using a bioinformation database [35, 36]. Based on the PubMed database, the regulated targets of these miRNAs and their possible functions in GC, breast cancer, lung cancer, and other cancers are listed in Table 1. Based on the sponge theory, hsa_circ_0005556 may act as a ceRNA to bind these miRNAs, which may take part in the regulation of GC progression. However, these predictions have not yet been verified. Next, we need to carry out a corresponding experiment to explore whether hsa_circ_0005556 regulates the function of GC by binding these sites.

The association between circRNAs and some clinicopathological features has a strong clinical value. Sufficient tumor resection is the key therapeutic factor for resectable GC [37, 38]. The main treatments for resectable GC include surgical resection and endoscopic resection. Surgical resection has been regarded as the gold standard because gastrectomy plus D2 lymphadenectomy has contributed to raise survival rates [3]. However, conventional surgery is associated with many complications, high costs, and extensive trauma. Despite the widespread use of laparoscopic techniques, they do not completely address these shortcomings. Comparatively, endoscopic treatment is a minimally invasive method that is safer and less costly in patients with EGC. A series of studies have proven that endoscopic resection is as effective as conventional surgery in EGC [39, 40]. However, the scope of endoscopic treatment is extremely strict, indicating that preoperative evaluation is crucial. Guidelines from Europe, Japan, and America all note that differentiation, invasion, and lymphatic metastasis are the key factors to assess the indication [41–43]. Current examination methods, including upper GI endoscopy and biopsy, endoscopic ultrasound, and CT, are not completely accurate. Particularly in judging lymph node metastasis, the CT method shows a high level of missed diagnoses because the detection rate of micrometastatic lymph nodes smaller than 10 mm in diameter is low [44]. Once a mistaken assessment is made, the incorrect choice in patient treatment will lead to an avoidable poor prognosis. In our research, there was a significant relationship between decreased hsa_circ_0005556 expression in GC and some clinicopathological factors, such as differentiation, lymphatic metastasis, and TNM stage (Table 1). The patients with a lower hsa_circ_0005556 level had worse degree of tumor differentiation and later TNM stage. Although there was no linear relationship between the hsa_circ_0005556 level and the number of lymph node metastases, the data showed that it can significantly distinguish between N(-) and N(+) (*p* = 0.01). These characteristics may help determine the diagnosis of EGC. As a result, hsa_circ_0005556 may contribute to the choice of treatment for GC, especially to determine whether the patient is suitable for endoscopic treatment instead of conventional surgery.

The developing standard of care in GC is evaluation of human epidermal growth factor receptor 2 (Her2), mismatch repair (MMR), and programmed cell death ligand 1 (PD-L1) status in tumors, with related targeted therapies informing medical and surgical management and studies for these proteins also being done on preoperative specimens [45, 46]. To advocate for the utility of circRNA in this current paradigm, we also tested whether the expression levels of these other markers are associated with hsa_circ_0005556 in the set of tumors. In our medical center, no tests were conducted for MMR and PD-L1 before 2015, and the test for Her2/neu was only carried out in 45 specimens. Patients were divided into two groups, Her2-negative and Her2-positive. We analyzed the hsa_circ_0005556 expression in the tissues of the two groups. The data were in accordance with the normal distribution. The results showed the hsa_circ_0005556 expression of the Her2-positive group was not significantly different from that of the Her2-negative group (group Her2-negative, *n* = 38, mean ± SD = 12.33 ± 1.47; group Her2-positive, *n* = 7, mean ± SD = 11.79 ± 0.93; *p* = 0.35). However, the number of sample is too small, especially for the Her2-positive group due to the low-Her2 positive rate ranging from 10% to 20%. In the future, we need to expand the sample for further study.

## 5. Conclusion

In summary, our data indicated that hsa_circ_0005556 expression is significantly downregulated in GC tissues. The downregulated expression level was correlated with differentiation, TNM stage, and lymphatic metastasis. Our research suggests that hsa_circ_0005556 may become a biomarker for GC. hsa_circ_0005556 may help to decide the method of treatment for EGC by judging the indications of endoscopic treatment.

## Figures and Tables

**Table 1:** The predicted miRNAs targeting hsa_circ_0005556 and their potential functions in tumors.

MicroRNA	Target	Tumor type	Function	Ref.
miR-548c-3p	HIF-1*α*, VEGF	Breast cancer	Proliferation, invasion, chemotherapy resistance	[16, 17]
	c-Myb	Glioma	Proliferation, migration	[18]
	ITGAV	Osteosarcoma	Proliferation	[19]
	——	Gastric cancer	Differential expression in HP(+) and HP(-)	[20]
miR-587	PPP2R1B	Colorectal cancer	Chemotherapy resistance	[21]
	——	Glioblastoma multiforme	Prognosis	[22]
miR-4739	*β*-Catenin	Gastric cancer	Proliferation, invasion, chemosensitivity	[23]
miR-125a-3p	TIM-3	Acute myeloid leukemia	——	[24]
miR-125a-3p	Fyn	Pancreatic ductal adenocarcinoma	Chemosensitivity	[25]
	MTA1	Lung cancer	Proliferation, migration, invasion	[26]
	CDK3	Lung cancer	——	[27]
miR-297	VEGFA	Oral squamous cell carcinoma	Proliferation	[28]

HIF-1*α*: hypoxia-inducible factor-1 alpha, VEGF: vascular endothelial growth factor, c-Myb: protooncogene c-Myb, ITGAV: integrin *α*v, TIM-3: T cell immunoglobulin and mucin-3, MTA1: metastasis-associated gene 1, CDK3: cyclin-dependent kinase 3, VEGFA: vascular endothelial growth factor A.

**Table 2:** Relationship between hsa_circ_0005556 expression levels (*Δ*Cq) and clinicopathological factors of patients with gastric cancer.

Characteristics	No. of cases (%)^∗^	Mean ± SD	*p* value
Age (years)			0.331
<60	28 (29.5)	11.88 ± 1.51	
≥60	67 (70.5)	12.27 ± 1.86	
Gender			0.655
Male	25 (26.3)	12.29 ± 1.73	
Female	70 (73.7)	12.11 ± 1.79	
Diameter (cm)			0.061
<5	42 (44.2)	11.78 ± 2.00	
≥5	53 (55.8)	12.46 ± 1.51	
Differentiation			**0.001**
Well	8 (8.4)	9.97 ± 2.87	
Moderate	22 (23.2)	12.09 ± 1.29	
Poor	65 (68.4)	12.45 ± 1.56	
Lymphatic metastasis			**0.039**
N0	33 (34.7)	11.53 ± 1.92	
N1	19 (20.0)	12.24 ± 1.25	
N2	12 (12.6)	12.13 ± 1.59	
N3	31 (32.6)	12.79 ± 1.76	
Invasion			0.383
T1&TIS	17 (17.9)	11.52 ± 2.49	
T2	12 (12.6)	12.21 ± 1.35	
T3	8 (8.4)	12.66 ± 2.01	
T4	58 (61.0)	12.26 ± 1.54	
Distal metastasis			0.779
M0	87 (91.6)	12.14 ± 1.77	
M1	8 (8.4)	12.32 ± 1.83	
TNM stage			**0.013**
I&II	42 (44.2)	11.66 ± 1.82	
III&IV	53 (55.8)	12.55 ± 1.63	
CEA			0.415
Positive	12 (12.6)	11.77 ± 3.05	
Negative	83 (87.4)	12.21 ± 1.51	
CA19-9			0.617
Positive	42 (44.2)	12.05 ± 2.00	
Negative	53 (55.8)	12.24 ± 1.57	

SD: standard deviation; CEA: carcinoembryonic antigen; CA199: carbohydrate antigen 19-9; 5 patients are not included due to incomplete clinicopathological information.

## Data Availability

(1) The microarray analysis data used to support the findings of this study have been deposited in the GEO dataset (https://www.ncbi.nlm.nih.gov/geo/query/acc.cgi?acc=GSE89143). (2) The quantitative reverse transcription-polymerase chain reaction (qRT-PCR) of hsa_circ_0005556 data used to support the findings of this study are included within the article.
